# Modeling eelgrass spatial response to nutrient abatement measures in a changing climate

**DOI:** 10.1007/s13280-020-01364-2

**Published:** 2020-08-13

**Authors:** Ivo C. Bobsien, Wolfgang Hukriede, Christian Schlamkow, René Friedland, Norman Dreier, Philipp R. Schubert, Rolf Karez, Thorsten B. H. Reusch

**Affiliations:** 1 GEOMAR Helmholtz Centre for Ocean Research Kiel, Marine Evolutionary Ecology, Düsternbrooker Weg 20, 24105 Kiel, Germany; 2 Rostocker Fracht- und Fischereihafen GmbH, Fischerweg 408, 18069 Rostock, Germany; 3 Leibniz-Institute for Baltic Sea Research Warnemünde, Seestraße 15, 18119 Rostock, Germany; 4 Institute of River and Coastal Engineering, Hamburg University of Technology, Denickestr. 22, 21073 Hamburg, Germany; 5 State Agency for Agriculture, Environment and Rural Areas Schleswig-Holstein (LLUR), Hamburger Chaussee 25, 24220 Flintbek, Germany

**Keywords:** Baltic Sea Action Plan, Climate change, Eutrophication, Scenario modeling, Species distribution model, *Zostera marina*

## Abstract

For many coastal areas including the Baltic Sea, ambitious nutrient abatement goals have been set to curb eutrophication, but benefits of such measures were normally not studied in light of anticipated climate change. To project the likely responses of nutrient abatement on eelgrass (*Zostera marina*), we coupled a species distribution model with a biogeochemical model, obtaining future water turbidity, and a wave model for predicting the future hydrodynamics in the coastal area. Using this, eelgrass distribution was modeled for different combinations of nutrient scenarios and future wind fields. We are the first to demonstrate that while under a business as usual scenario overall eelgrass area will not recover, nutrient reductions that fulfill the Helsinki Commission’s Baltic Sea Action Plan (BSAP) are likely to lead to a substantial areal expansion of eelgrass coverage, primarily at the current distribution’s lower depth limits, thereby overcompensating losses in shallow areas caused by a stormier climate.

## Introduction

Marine coastal ecosystems are suffering particularly from ongoing global change, including ocean warming, acidification, deoxygenation, and eutrophication (Rabalais et al. 2009), in addition to enhanced storminess and wave energy impinging on shorelines (Young and Ribal 2019). In order to secure sustainable use of coastal ecosystems, and at the same time protect and preserve marine habitats and ecosystem functioning for future generations, integrated ecosystem-based management strategies and concepts are needed (Fernandino et al. 2018). Any effective marine spatial planning includes systematic conservation approaches that depend on reliable information on the distribution of species or valuable habitats, as well as understanding how ecosystems will respond to anthropogenic pressure. In this context, species distribution models have become highly useful and cost-effective tools in coastal marine management and conservation planning (Fyhr et al. 2013).

At the European level, several legislative frameworks such as the Water Framework Directive (EC 2000) and the Marine Strategy Framework Directive (EC 2008) have been adopted aiming to achieve a ‘good environmental status’ (GES) in coastal and open ocean waters. These directives demand coordinated measures to promote ecosystem recovery and indicate the need for assessing the benefits of environmental rectification. The protection of the Baltic Sea, one of the largest semi-enclosed brackish water seas in the world, is the target of the Commission for the Protection of the Marine Environment of the Baltic Sea (HELCOM). HELCOM has established the Baltic Sea Action Plan (BSAP; HELCOM 2007), an ambitious management program aiming to reduce nutrient pollution and to reverse ecosystem degradation of the Baltic aquatic environment. The BSAP committed each member state to nutrient input ceilings to restore the Baltic marine environment by 2021 (Backer et al. 2010). It is less well established, however, whether the proposed reduction goals will be sufficient to result in significant recoveries of valuable ecosystems that have declined in the past, such as eutrophication sensitive seagrass beds that are one prime target for coastal conservation effort. Being a polyphyletic group of marine flowering plants, seagrasses are the foundation of one of the most valuable ecosystems in shallow coastal waters (Nordlund et al. 2016). Seagrasses improve water quality and clarity, foster sediment stability and thus enhance coastal protection, and bind and sequester nutrients and carbon (Moore 2004; Ondiviela et al. 2014; Duarte and Krause-Jensen 2017). Due to their ability to accumulate and store organic carbon in the sediments over millennial time scales, seagrass meadows are significant “blue carbon” sinks (Fourqurean et al. 2012). However, seagrass beds are facing significant anthropogenic threats as a result of eutrophication and climate change (Duarte et al. 2018). Currently, seagrasses are in decline, with global annual losses of about 7% since 1990 (Waycott et al. 2009) although losses in some European regions have come to a halt (de los Santos et al. 2019), prompting the question how meadows can be promoted to recover.

We here couple climate-forcing projections of sea state and biogeochemical model projections of water turbidity with species niche modeling to forecast the future spatial distribution of the eelgrass (*Zostera marina*) on local scales. Input variables represent ecological key predictors, which were parameterized to the International System of Units to simplify future projections. Our main objective was to quantify *Zostera marina*’s spatial response to nutrient mitigation efforts according to the BSAP reduction targets in combination with future climate change and resulting sea state scenarios.

## Materials and methods

### Study site

**Fig. 1:**
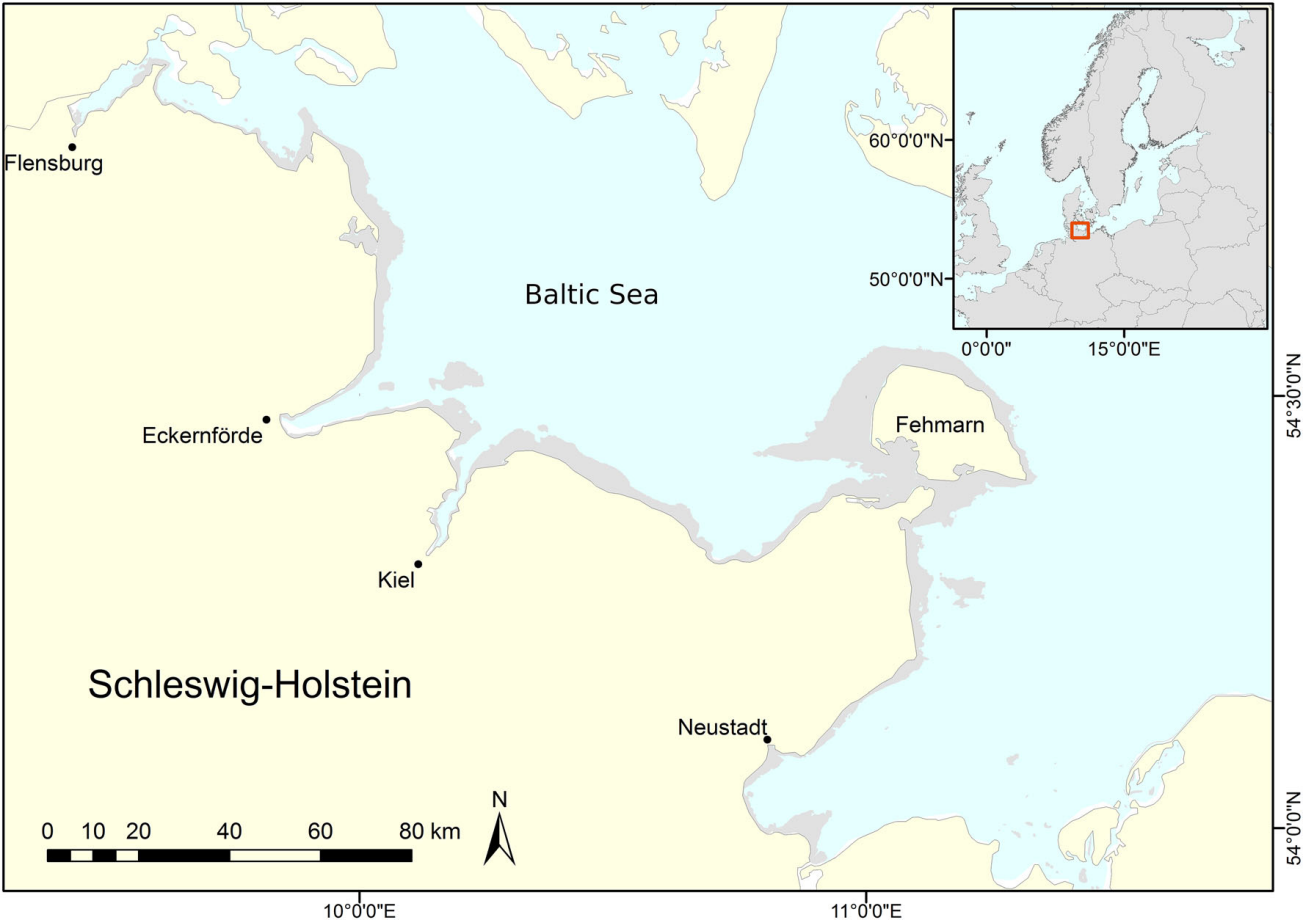
Baltic Sea coast of Schleswig-Holstein with the island of Fehmarn. Water depths < 12 m are marked with gray color

The study region comprises the western part of the Baltic Sea coast in northern Germany (i.e., the federal state’s Schleswig-Holstein eastern coastline). Relatively shallow bays and one island (Fehmarn) characterize this region (Fig. 1).

Due to prevailing westerly winds and the enclosed character of the coast, wave exposure is mostly slight and significant wave heights rarely exceed 3 m (Petterson et al. 2018). Shallow surface water salinity ranges from ~ 8 to ~ 18 PSU depending on inflow events of fully saline water from the North Sea, as well as location and depth (Franz et al. 2019). Water currents are weak overall except for narrow fjords, when strong winds induce rapid sea-level changes. The sea bottom primarily consists of sandy-to-muddy sediments, partly interspersed with boulders, cobbles, and gravel. Bedrock is absent throughout the study area. Eelgrass is the dominant vegetation type along the coastline, where it inhabits water depths between 1 and 8 m (Schubert et al. 2015).

### Model approach and response variables

We applied the software GRASP (generalized regression analysis and spatial prediction, Lehmann et al. 2002) within the statistics package R (R Development Core Team 2008) to calculate generalized additive models (Hastie and Tibshirani 1990). GRASP allows for generating species response curves showing the effect of the applied environmental gradients. The input data base of 7 150 geographically referenced presence vs. absence records was obtained from extensive eelgrass video transect mappings in 2010 and 2011 (Schubert et al. 2015). The distribution model covers the study area from the shoreline down to 12 m water depth (Fig. 1) with a grid size of 100 m, comprising a total area of 828 km^2^. For each grid point the model calculated the probability of eelgrass occurrence (values ranging from 0 to 1). The balanced prevalence with the same numbers of presence and absence records allowed for translating the probabilities of eelgrass occurrence directly to probabilities of plant encounter (equivalent to percent eelgrass coverage) without further modification (Liu et al. 2005). Water depths for model calculations were derived from an array of diverse digital elevation models. First, a digital bathymetric map based on airborne LiDAR measurements (Light Detection and Ranging) from between 2014 and 2016 (1 m spatial resolution), delivered values for the shallowest coastal regions from 0 to 2.5 m. This data set and a digital shoreline produced from aerial orthographic photos taken in 2013 were provided by the State Agency for Coastal Protection, National Park and Marine Conservation Schleswig–Holstein (LKN.SH). In places where no LiDAR depth data were available, we used interpolated sonar depth measurements of the Federal Maritime and Hydrographic Agency (BSH, Germany) between 1982 and 2004. This bathymetric layer covers water depths from 1.0 to 34.0 m with a horizontal resolution of 10 to 50 m. Some still missing depth data, primarily in places deeper than 10 m, were taken from a third bathymetric map with 50 m spatial resolution, which was provided by the State Agency of Agriculture, Environment and Rural Areas Schleswig–Holstein.

### Environmental predictors

We designated light availability for eelgrass and wave-generated water current at the bottom as the most important environmental variables regulating the spatial distribution of eelgrass. While the environmental variable “salinity” had been tested in earlier model runs based on the same set of eelgrass distribution input data (Schubert et al. 2015), it does not significantly increase the predictive power of the eelgrass model and was therefore excluded from the current model.

#### Light availability

Photosynthetic photon flux density (PFD, μmol photons m^−2^ s^−1^) available for plant growth was calculated for a depth of 0.5 m above sea floor (roughly corresponding to the top of the eelgrass canopy) using Beer’s law *I*_*z*_ = *I*_0_ × *e*^−*k*×*z*^ (Kirk 1994), where *I*_*z*_ denotes the irradiance at water depth *z*, *I*_0_ the surface irradiance, and k the light attenuation coefficient. Mean monthly surface solar irradiance was taken from the European Commission’s Photovoltaic Geographical Information System PGIS (http://re.jrc.ec.europa.eu/pvgis/apps4/pvest.php?lang=de&map=europe), based on satellite measurements between 1998 and 2011 (Huld et al. 2012). For simplicity, the surface irradiance of a representative position in the model region (54°28′58″ N, 10°21′36″ E) was used over the entire model grid. Irradiance to PFD conversion was achieved by multiplying the solar irradiation (W m^−2^) with a factor of 4.15 (Morel and Smith 1974). The attenuation coefficient k was estimated from modeled Secchi depths (SD) taken from the coupled biogeochemical and hydrological model ERGOM-MOM (Friedland et al. 2012). No attempt was made to account for variable surface roughness during different wind situations, i.e., the model is based on plane water surface conditions (no wind-induced roughness). Since according to Kirk (1994) nearly 6% of the incident light is reflected from the water, some overestimation ensues.

To obtain estimates for future changes of PFD in the context of nutrient discharge regulation measures, we ran simulations with ERGOM-MOM. This model encloses the whole Baltic Sea, but only data points at the study area (with a grid resolution of 1 nautical mile) were used. ERGOM-MOM incorporates the inorganic nutrients ammonium, nitrate, and phosphate, which enter the Baltic Sea via waterborne loads and atmospheric deposition, as well as three functional phytoplankton groups, and one bulk group of zooplankton and detritus (Neumann 2000). Light attenuation was calculated depending on phytoplankton and detritus concentrations (Friedland et al. 2012). Modeled Secchi depths were validated using empirical observations obtained from the responsible environmental authorities and institutions, i.e., the State Agency for Agriculture, Environment and Rural Areas. Emission scenario A1B of the International Panel on Climate Change (IPCC) was used for the nutrient scenario assessment. The A1B scenario belongs to the A1 greenhouse gas emission scenario family presented in the Special Report of Emission Scenarios of the IPCC. The A1 family is characterized by rapid economic growth, a global human population that peaks in mid-century and then gradually declines, a quick spread of new and efficient technologies and a convergent world (globalization). The A1 scenario splits into three groups that describe alternative directions of technological development with A1B achieving a balance between fossil and non-fossil energy sources (Nakićenović et al. 2000).

We ran two simulations with different nutrient load scenarios. The first simulation assumes a full implementation of the HELCOM nutrient input targets (BSAP scenario). The maximal allowable inputs given by the revised Baltic Sea Action Plan (HELCOM 2013) are heeded from 2021 on, after between 2012 and 2020 the nutrient loads were reduced linearly. The second scenario (business as usual, BAU scenario) kept the nutrient loads from 2012 onwards constant on the level of the BSAP reference period (1997–2003, HELCOM 2013). The baltic-wide difference between the two scenarios of the annual loads amounts to approximately 118 kt nitrogen and 15 kt phosphorus.

#### Hydrodynamic exposure to wave-generated orbital currents

Hydrodynamic exposure was represented by wave-generated maximum orbital velocity (MOV) at the sea floor, which depends on the local sea state. It was calculated as a function of simulated significant wave height (*H*_m0_), mean wave period (*T*_m02_), and wave length (*L*) for intermediate water depths (*z*) according to linear wave theory. We applied the criterion that the maximum wave height (*H*_max_) cannot exceed the local water depth (*d*) at a certain point (*H*_max_/*d* < 1). Based on the widely used assumption in coastal engineering praxis that *H*_m0_ ~ *H*_1/3_, we calculated the maximum wave height as *H*_max_ = 1.86 × *H*_m0_ (under the assumption of a Rayleigh distribution and *N* = 1 000 waves) and the maximum wave period as *T*_Hmax_ = 0.83 × *H*_max_ + 3.17 (Schlamkow pers. comm.). The necessary sea state data were derived from the WBSSC (Western Baltic Sea State Climate Version) wave model (Dreier et al. 2014). This model is based on the third-generation spectral wave model SWAN (Simulating Waves Nearshore, Booij et al. 1999). It includes (i) bathymetric data of the western Baltic Sea with a horizontal resolution of ~ 1 km, (ii) hourly wave spectra along the northern and eastern boundaries from the spectral wave model WAM (The Wamdi Group 1988) for the whole Baltic Sea (Groll et al. 2017), and (iii) hourly wind data from the regional climate model Cosmo-CLM (Lautenschlager et al. 2009). The Cosmo-CLM model was forced from the global AOGCM ECHAM5/MPI-OM with observed anthropogenic emissions (twentieth century run: 1961–2000) and with two of the future emission scenarios used within IPCC-Assessment Report 4 (twenty-first century run: 2001–2100), namely A1B (global economic emission scenario) and B1 (global environmental emission scenario) (Nakićenović et al. 2000). Each of the two emission scenarios were simulated twice, starting with slightly different initial conditions (different years in the past) and resulting in four possible model realizations. The use of different realizations of the same emission scenario is a common approach used by the climate modeling community to account for the internal variability of the climate system. Consequently, four long-term wave projections (two emission scenarios with two realizations each) were available as input for the eelgrass distribution model (Dreier et al. 2014).

### Reference state and modeling strategy

The species distribution model relates field observations of eelgrass occurrence to environmental variables. As eelgrass mapping was performed in the summers of 2010 and 2011, the latter year was taken to represent the reference status of eelgrass distribution. The eelgrass distribution was assumed to be in equilibrium with its environment with presence in all suitable locations and absence in locations with unsuitable environmental conditions. The changes of the predictor variables are projected for future model scenarios including two nutrient load scenarios and four future wave scenarios. This implies that the same set of future wave data is applied once to each of the two nutrient load scenarios. The eelgrass distribution model covers a time period of 60 years (2007–2066). We specified twelve 5-year time slices (2007–2011 as the baseline, followed by 2012–2016, 2017–2021, etc., until 2062–2066) to model the future eelgrass distribution. Within each of these twelve intervals, a 5-year mean of the predictor variables was established. On the base of the twelve time intervals this approach finally results in 92 model runs comprising four models for the base line of eelgrass distribution (2007–2011) and eight model runs for each of the future time intervals, accounting for 88 models.

To visualize the changes in the future eelgrass occurrence, we prepared maps showing the differences of the modeled probability of eelgrass occurrence between the reference period 2007–2011 and the 2061–2066 time interval for each of the model scenarios (Table 1).

**Table 1:** Differences in 5-year means (± SD) of photon flux density (PFD), maximum orbital velocity (MOV), and probability of eelgrass occurrence (PEO) between the 2007–2011 and the 2061–2066 time intervals as calculated with the eelgrass distribution model for the study area. The BSAP scenario represents the complete implementation of the Baltic Sea Action Plan nutrient reduction targets. The BAU scenario represents nutrient loads according to the BSAP reference period (1997–2003). The four MOV scenarios account for the greenhouse gas emission scenarios A1B and B1 of the Intergovernmental Panel on Climate Change each in two random realizations. Finally, the eelgrass models combine both approaches

	Scenario	Mean ± SD	Min	Max
PFD (µmol photons m^−2^ s^−1^)	BSAP	8.2 ± 5.6	− 2.6	45.5
	BAU	0.6 ± 2.2	− 10.2	9.4
MOV (m s^−1^)	A1B_1	− 0.012 ± 0.012	− 0.66	0.001
	A1B_2	− 0.008 ± 0.014	− 0.56	0.138
	B1_1	0.007 ± 0.007	− 0.005	0.381
	B1_2	− 0.0004 ± 0.011	− 0.377	0.241
PEO	BSAP/A1B_1	0.045 ± 0.039	− 0.011	0.287
	BSAP/A1B_2	0.040 ± 0.037	− 0.029	0.287
	BSAP/B1_1	0.028 ± 0.032	− 0.06	0.238
	BSAP/B1_2	0.036 ± 0.035	− 0.042	0.282
	BAU/A1B_1	0.012 ± 0.017	− 0.065	0.109
	BAU/A1B_2	0.008 ± 0.016	− 0.06	0.112
	BAU/B1_1	− 0.003 ± 0.012	− 0.075	0.048
	BAU/B1_2	0.003 ± 0.013	− 0.067	0.079

To attribute spatial distribution patterns and areal changes to each of the predictor variables, we also pursued a ceteris paribus approach, by keeping one of the variables (PFD resp. MOV) constant. Temporal trends of the total eelgrass area were charted using 30-year running means.

### Prediction accuracy

Model accuracy was ascertained in a five-fold cross-validation procedure by resampling, combined with threshold-independent receiver-operating-characteristic analysis (ROC, Fielding and Bell 1997). For each of the five iterations within the validation process a subset of the data was withhold during model building and used as test data. Calculating the area under the ROC curve (AUC) provides a measure of the model’s discriminatory capacity. With AUC values ranging from 0.5 to 1.0, a value of 0.5 denotes no, 0.7 low, 0.7 to 0.8 acceptable, and 0.8 to 0.9 excellent discriminative abilities (Hosmer and Lemeshow 2000).

## Results

**Fig. 2:**
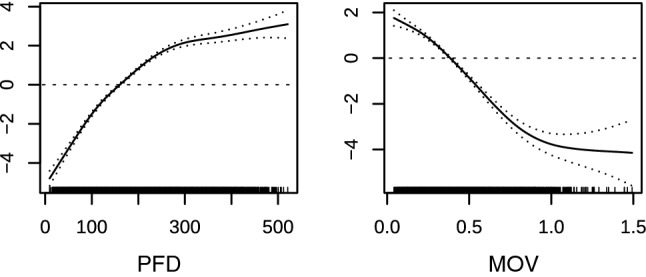
Response of *Zostera marina* to PFD (Photon Flux Density, μmol photons m^−2^ s^−1^ at 0.5 m above the sea floor) and MOV (maximum orbital wave velocity, m s^−1^ at the sea floor). The range of both predictor variables is displayed on the *x*-axis while the *y*-axis shows the probability of eelgrass occurrence (on a logit scale). The horizontal dashed line marks the probability of occurrence of 0.5. Small ticks above the *x*-axis represent single observations; the semi dashed lines indicate the 95% confidence interval limits around the response curve

Our eelgrass distribution model features acceptable prediction accuracy with a mean five-fold cross-validated Area Under Curve value of 0.77 ± 0.01 (mean ± SD) over all emission scenarios. Photon flux density PFD and maximal orbital water velocity MOV proved to be suitable environmental variables for predicting eelgrass occurrence, with 57.2% and 42.7% contribution, respectively. The eelgrass response curves show opposite curve characteristics across the gradients of light and wave-induced water currents (Fig. 2).

Eelgrass response to increasing light intensities shows a saturation-type response with a linear positive slope until 200 μmol photons m^−2^ s^−1^ are attained. In contrast, eelgrass distribution steeply decreases with increasing orbital water movement. The response curve declines until it flattens at > 1 m s^−1^, while water currents < 0.4 m s^−1^ are indicative of eelgrass presence.

### Model scenarios

**Fig. 3:**
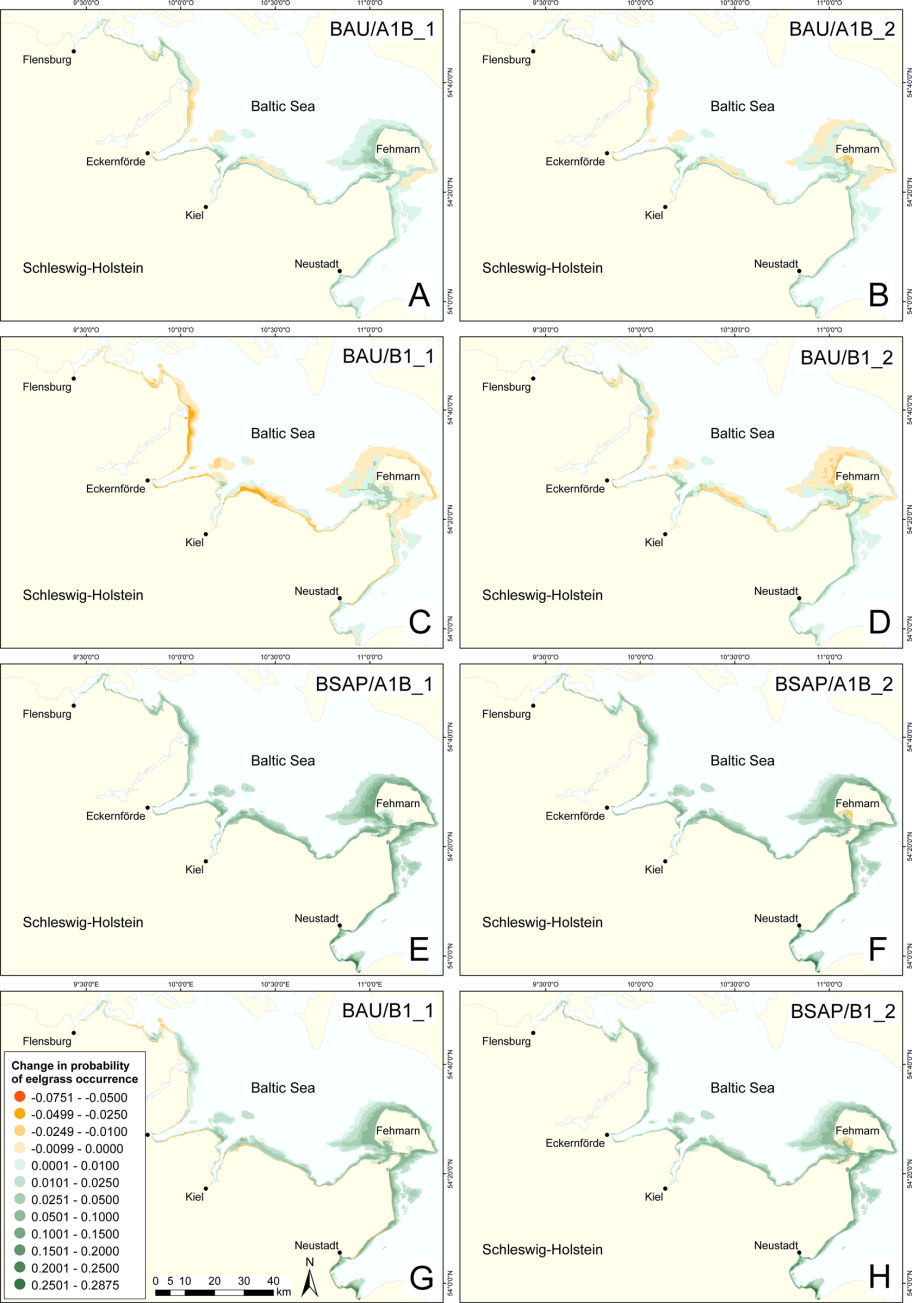
Changes in probability of eelgrass occurrence at the Baltic Sea coast of Schleswig-Holstein until 2066. BAU represents the business as usual nutrient regime, referring to constant nutrient loads on the level of the BSAP reference period (**a**–**d**) while BSAP represents the full implementation of the Baltic Sea Action Plan nutrient reduction targets (**e**–**h**). Both regimes are combined with climate-related variation of wave power according to the A1B and B1 greenhouse gas emission scenarios of the Intergovernmental Panel on Climate Change, each in two random realizations

The different model runs predict widely divergent future eelgrass distribution patterns. Compared to the reference period (2007–2011), the mean eelgrass distribution area increases by 16.3 ± 2.9% (mean ± SD) in the Baltic Sea Action Plan model scenarios (BSAP) until 2066, corresponding to an overall areal increase of 30.1 ± 5.3 km^2^ (mean ± SD) (Fig. 3e–h). Depending on the modeled wave scenarios and on the selected time slice, increasing or decreasing maximum orbital velocities either strengthens or weakens eelgrass expansion.

The most significant increases in eelgrass occurrence of 19.9% (36.6 km^2^), 17.7% (32.8 km^2^), and 15.6% (28.8 km^2^) were found based on the BSAP scenario in combination with climate wave scenarios A1B_1, A1B_2, and B1_2, respectively (Fig. 3e–h), when nutrient reduction occurs along with decreasing wave energy (i.e., maximum orbital velocity, MOV values) (Table 1). Likewise, but less prominent, an increase of 11.9% (22.3 km^2^) was observed within the BSAP/B1_1 scenario (Fig. 3g) when nutrient abatement coincides with increasing MOV (Table 1).

The overall changes in eelgrass occurrence under business as usual range from − 1.3% in the B1_1 to +1.1% in the B1_2 and from +3.6% in the A1B_2 to +5.3% in the A1B_1 wave scenario until 2066 (Fig. 3c–a). Negative impact of higher MOV (+3.9 ± 1.8% until 2066; mean ± SD) on the overall eelgrass distribution is most apparent in the B1_1 wave scenario (Fig. 3c), where the total eelgrass coverage decreases by 2.5 km^2^. This scenario is characterized by wide-ranging spatial variation for all of the open exposed coastlines as well as the shallow sheltered bays, but also for deeper eelgrass meadows. While the overall effect on the predicted eelgrass occurrence is comparably low in the BAU scenario in combination with the A1B_1, A1B_2, and B1_2 wave scenarios (Fig. 3a, b, and d) the modeling results still indicate potential sensitive areas. These regions are sheltered areas such as the Orth Bay in the southwest of the island of Fehmarn and Gelting Bay eastwards of Flensburg, but also exposed sections of the outer coastal strip (Fig. 3).

### *Ceteris paribus* modeling

When keeping the maximum orbital velocity (MOV) constant until 2066, changes in photon flux density (PFD) cause the mean eelgrass distribution in the Baltic Sea Action Plan scenario (BSAP) to increase by 15.1 ± 0.5% (mean ± SD), while being nearly unaffected under business as usual conditions (0.9 ± 0.05%; mean ± SD). If on the other hand PFD is kept constant, the mean eelgrass distribution is not changing significantly (1.2 ± 2.5%; mean ± SD), but the large confidence range indicates MOV to tip the scales to a positive or negative outcome. Finally, changes in PFD contribute 93% to the total areal change in the BSAP scenarios, whereas changes in MOV potentially contribute 83% to the variation. Conversely, MOV contributes 57% to the total eelgrass areal change in the BAU scenarios, whereas PFD contributes only 2% to the variation.

### Total eelgrass area

**Fig. 4:**
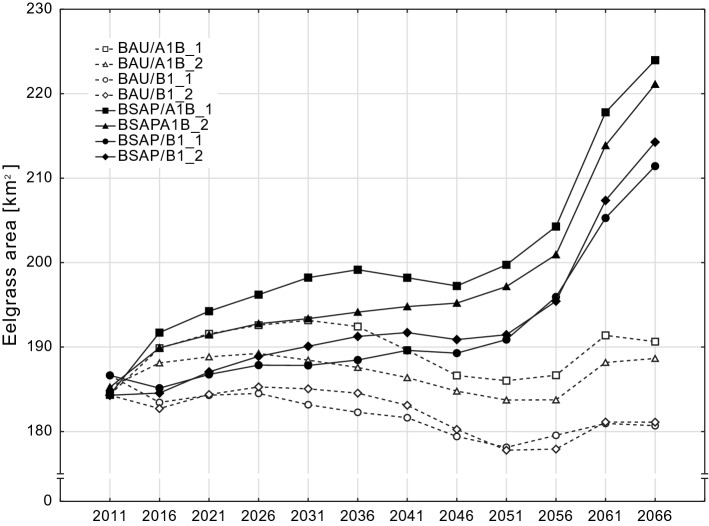
Modeled overall eelgrass area for the Baltic Sea coast of Schleswig-Holstein until 2066 as a function of nutrient reduction scenarios and climate-related sea state conditions. Data points represent running means over 30 years. BSAP: Implementation of the Baltic Sea Action Plan nutrient reduction targets. BAU: nutrient loads according to the BSAP reference period. A1B and B1 represent the greenhouse gas emission scenarios according to the Intergovernmental Panel on Climate Change each in two random realizations

In contrast to business as usual, the overall eelgrass area expanded considerably under nutrient abatement as required by the Baltic Sea Action Plan (BSAP). Substantial increase will occur over the last 15 years with a time lag of about 30 years after the full implementation of the nutrient reduction targets in 2021 (Fig. 4). The variation within both nutrient reduction scenarios (BSAP and BAU) is exclusively attributable to changes in MOV and therefore a result of the random variability in the four realizations of the emission scenarios.

### Predicting eelgrass depth distribution

**Fig. 5:**
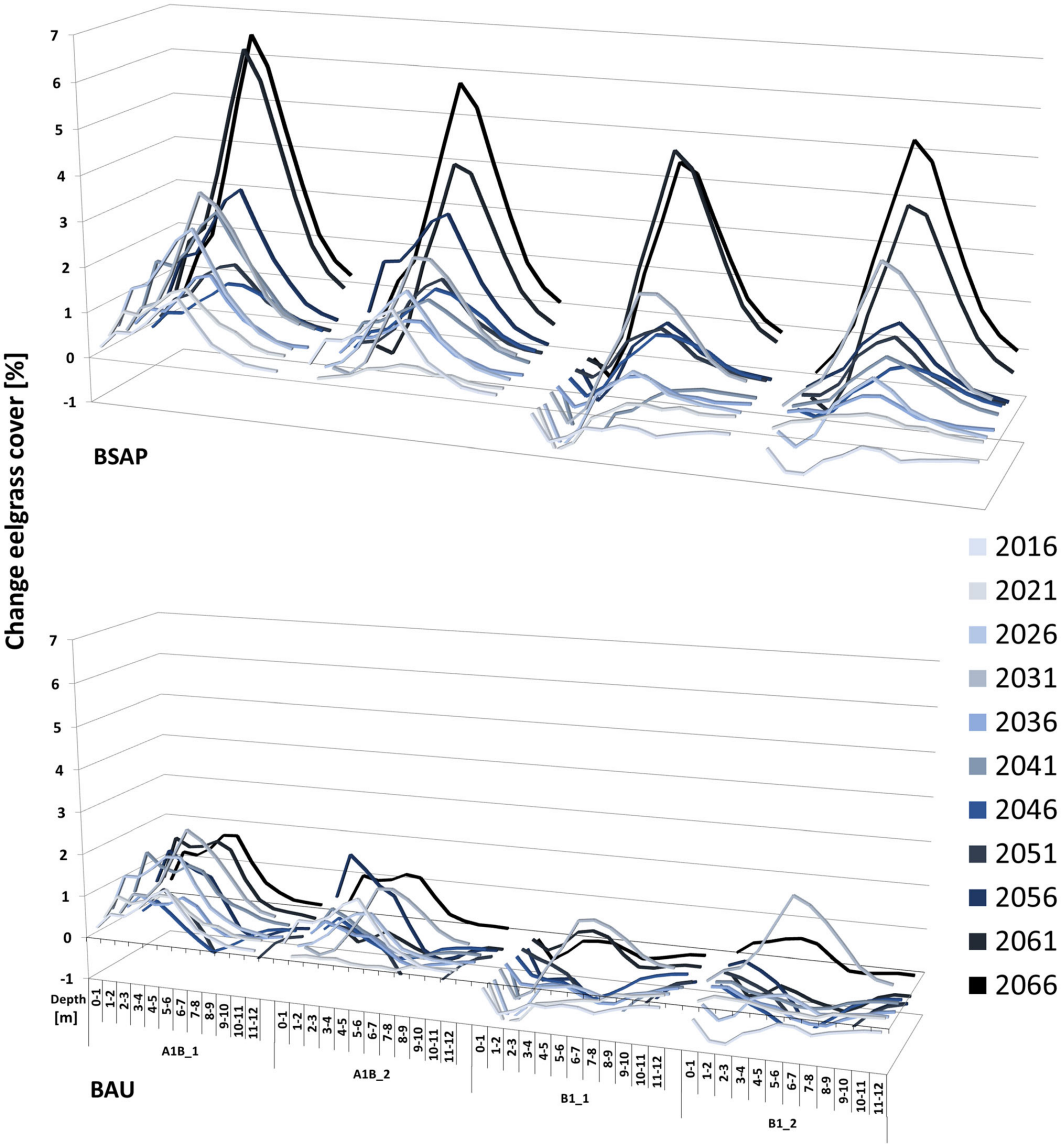
Percentual changes in eelgrass coverage as a function of water depth dependent on nutrient reduction scenarios and climate-related wave scenarios. BSAP: Implementation of the Baltic Sea Action Plan nutrient reduction targets. BAU: nutrient loads according to the BSAP reference period (1997–2003). A1B and B1 represent greenhouse gas emission scenarios according to the Intergovernmental Panel on Climate Change each in two random realizations

The applied predictor variables modulate the eelgrass distribution differently in different depth zones. Improved light supply leads to increasing eelgrass predictions, especially at greater depth, such that within the BSAP models the probability of occurrence rises considerably between 4 and 8 m, with a maximum at 5 to 6 m (Fig. 5). Conversely, high variation is caused by MOV in the different climate wave scenarios at water depths < 4 m. Below 8 m the variability decreases continuously to minimum levels at a lowest depth of 11–12 m (Fig. 5).

## Discussion

Our scenario modeling indicates that nutrient abatement according to the Baltic Sea Action Plan (BSAP scenario) enhances the occurrence of *Z. marina* along the Baltic coast of northern Germany. Remarkably, the areal expansion, which is expected as a benefit of nutrient reduction, more than compensates potential areal loss from stormier conditions and higher wave energy under anticipated climate scenarios. Assuming the BSAP scenario enhanced light levels in the eelgrass meadows’ growing zone shift the suitable habitat conditions to greater depth, which induces a net increase of eelgrass coverage.

Hence, our results highlight the paramount significance of nutrient reduction measures to the recovery of threatened and ecologically valuable eelgrass meadows. Further, they underline that the specified BSAP reduction targets suffice to initiate environmental improvement. Therefore, the implementation of the BSAP targets should be pursued persistently, the more so as latest findings indicate that nutrient inputs into the Baltic Sea are on the rise again after a phase of conspicuous decline (Murray et al. 2019; Olesen et al. 2019).

Assuming a business as usual (BAU) scenario, the ERGOM-MOM model projections suggest no significant deterioration of the underwater light conditions for the study region until 2066 (Table 1). Consequently, the eelgrass model indicates no substantial change of the overall eelgrass coverage, which suggests that we are presently looking at some kind of equilibrium distribution that will likely not further deteriorate. Like in most other regions of the Baltic Sea, the eelgrass meadows of the German Baltic Coast (Schleswig–Holstein) declined to nearly 50% of their historical distribution in the 1960s mainly as a result of eutrophication effects (Boström et al. 2014; Schubert et al. 2015). While some coastal regions still suffer losses without any signs of reaching equilibrium or trend reversal (Moksnes et al. 2018) recent studies reported that the rate of seagrass losses slowed down for most of the European seagrass species and fast growing species even recovered in some locations (de los Santos et al. 2019). Actual monitoring data for the Baltic Sea coast of Schleswig-Holstein confirm the same overall maximum depth limits of eelgrass or even slightly increasing trends over the last 10 years (Karez pers. com.), which supports the idea of a current equilibrium distribution. These findings coincide with observations of Riemann et al. (2016) for Danish eelgrass meadows.

The variations in eelgrass distribution patterns do not only result from changes in wave height, but also from changes in wave direction (Dreier et al. 2014). Maximum orbital velocity (MOV) primarily affects the shallow distribution limit of eelgrass with only minor impact on the size of the overall distribution area. Within the B1_1 climate scenario, however, the model runs indicate substantial losses of shallow eelgrass meadows along the entire coastline across both nutrient regimes. Thus, more frequent and more violent storm events in the future may weaken the eelgrass’ potential for coastal erosion control (Ondiviela et al. 2014).

The species distribution model reproduces the ecophysiological tenet that light determines the macrophytes’ lower depth distribution, while physical exposure restricts the upper depth limit (Krause-Jensen et al. 2003). Assuming the BSAP scenario and hence more translucent water conditions, eelgrass distribution primarily increases at the current lower depth limit of 4 to 8 m (Schubert et al. 2015). In contrast, changes in wave energy, reflected in our chosen variable MOV, primarily affect eelgrass occurrence in 1 to 2 m depth, which coincides with the current minimum depth distribution in the study area.

Reassuringly, our modeling results closely match with known ecophysiological data on eelgrass light requirements (Lee et al. 2007) and tolerable water currents (Koch 2001). The photon flux density (PFD) response curve indicates a positive correlation of eelgrass occurrence with light intensities (Fig. 2) and with levels above 150 μmol photons m^−2^ s^−1^ (corresponding to about 25% of the solar surface irradiation) contributing positively to model predictions. This value fits into the range of eelgrass light compensation points calculated for whole plants (Lee et al. 2007) and coincides with the minimum light requirement for eelgrass, which varies between 18 and 29% of the surface irradiance (Krause-Jensen et al. 2011). The response curve for PFD flattens above 350 μmol photons m^−2^ s^−1^ (Fig. 2), in turn coinciding with eelgrass light saturation points for photosynthesis that are calculated for whole plants (Lee et al. 2007). The response curve for maximum orbital velocity indicates negative correlation with bottom water velocity, and the modeled habitat characteristics for eelgrass are less suitable if maximal water movement exceeds 0.4 m s^−1^. This threshold seems realistic for the study area. Generally, *Z. marina* can tolerate maximum current velocities of up to 0.5 to 1.8 m s^−1^ (Koch 2001). Fonseca and Kenworthy (1987), however, reported 0.5 m s^−1^ as a critical limit for the persistence of *Z. marina* meadows. In contrast to the nutrient reduction scenario, the overall eelgrass area in the BAU scenario remains nearly constant but shows an oscillating pattern. This wave-like pattern accounts for the interannual variability, which leads in the 3D model to increased chlorophyll concentrations and decreased Secchi depths around the year 2040. This increase is in agreement with previous studies, e.g., Friedland et al. (2012), who reported an increase of summer chlorophyll-*a* in the western Baltic Sea due to climate change, even if nutrient inputs stay on recent levels. The predicted overall eelgrass increases in the nutrient reduction scenarios are not evenly distributed over the modeled time period. Our model indicates that the nutrient abatement will improve eelgrass growth and abundance with a considerable delay of about 30 years. The time delay could be explained by the nutrient residence times in the Baltic Sea, as Radtke et al. (2012) estimated nutrients to stay at least 30 years in the Baltic Sea. But recovery times of submerged aquatic vegetation after cessation of nutrient inputs often take even longer and may vary from several years to nearly a century (McCrackin et al. 2016). Dispersal by seeds and rafting shoots to particular locations may not be efficient, and once first colonizers arrive, it may take some time until critical threshold densities are exceeded that allow for continual growth of the colonizing patches (Olesen and Sand-Jensen 1994). For temperate eelgrass meadows in Danish waters, Riemann et al. (2016) reported a similar time delay of increasing depth limits after 25 year of consistent nutrient mitigation measures. Light and water currents have been identified as major factors most often regulating the survival and the depth distribution of seagrasses (de Boer 2007). Therefore, we suggest the principal model approach to be directly applicable to other marine macrophyte species or in other regions of the world. Essential requirements for model setup, however, include extensive seagrass distribution data combined with local biogeochemical und hydrodynamic future model projections. Importantly, predictive variables of the spatial distribution model have to be parameterized in such a way that they can be coupled simultaneously to a climate-forcing model and to a biogeochemical ecosystem model.

The model calculates suitable habitat conditions (referring to light and wave-induced water currents as most important variables) on the basis of field observations and translates future variations of these conditions to areal changes (expansion or retreat) of the eelgrass distribution. We disregarded some restrictions or physical constraints that might possibly limit eelgrass expansion (Kuusemäe et al. 2016), especially, we excluded salinity from the current model because it did not significantly increase the predictive power of the model as tested in previous model runs (Schubert et al. 2015), which comes as no surprise as *Z. marina* is well adapted to low salinity and tolerates large salinity variations (Boström et al. 2014). Salinity as a regulating factor for *Z. marina* distribution becomes important in estuaries, freshwater-influenced lagoons, and brackish seas (oligohaline < 5 psu) where eelgrass is living at the edge of its salinity tolerance, while in our area, salinity is never below 8 psu. We recommend, however, including salinity to seagrass distribution models within areas where salinity is expected to reach the tolerance levels of the respective seagrass species.

Another process that may be given more consideration is the sediment-light feedback mechanism (van der Heide et al. 2011). Typically, dense seagrass meadows attenuate water currents and stabilize sediments; thereby they promote sedimentation and reduce resuspension of particles, which results in clearer water and more suitable growth conditions. At the moment, there is no stabilization feedback from eelgrass on the sediments included in the 3D-model (ERGOM-MOM), which was used to estimate the future development of light attenuation. It is well known that losses of seagrass, however, may trigger a regime shift to a more turbid state characterized by wind-driven resuspension of sediments that are no longer stabilized by eelgrass and therefore cause unsuitable light conditions for eelgrass colonization (Maxwell et al. 2016). Regime shifts to alternative stable states may prevent natural recovery of seagrass and make restoration quite difficult (Moksnes et al. 2018).

Biotic interactions may also be important environmental factors regulating the distribution of seagrasses (Nakaoka 2005). The loss of large predatory fish in the northern Kattegat, for example, induced complex trophic cascades affecting the survival of eelgrass due to light deficiency (Moksness et al. 2008; Casini et al. 2009). Including biotic interactions in addition to purely abiotic parameters that determine seagrass distribution is a major challenge for future research (Dormann et al. 2018). Further, major remaining gaps in our knowledge are in the field of effects of ocean warming. Increasing frequencies of heatwaves have shown to cause large mortality among eelgrass in the western Baltic Sea (Reusch et al. 2005) and their effects can be exacerbated due to increased water turbidity and anoxia (Moore and Jarvis 2008). We currently lack sufficient data to translate the expected rapid warming of Baltic Sea surface waters into eelgrass growth and distribution responses. Future sea level rise is another variable that needs further work, as it would effectively increase the actual bathymetric depth of up to 1 m, shifting the future distribution of eelgrass nearer to the present shorelines and requiring a shoreward distributional shift of some meadows occurring under very shallow slopes by hundreds of meters.

## Conclusion

We combined species distribution modeling with an ecosystem nutrient model and a climate forced wave forecasting model, which allowed us to project the spatial response of eelgrass to nutrient management measures in combination with emerging climate change and associated increase of wave energy. Our key result is that meeting nutrient reduction targets of the HELCOM Baltic Sea Action Plan would allow eelgrass to expand considerably and mostly in the vicinity to already existing eelgrass occurrences. We therefore do not anticipate that over decadal time scales dispersal limitation would delay recolonization much. The areal expansion and the increasing expansion into depth are likely to improve the ecological status of the German Baltic coastal water bodies as required by the European Water Framework Directive (EC 2000), which aims to achieve a “good ecological status” of the European surface waters.

Arguably, the exact projected distributional changes in eelgrass should not be taken at face value, but rather viewed as possible outcomes of divergent anticipated climate and ecosystem scenarios. The modeled expansion of eelgrass under ongoing nutrient abatement in our study nevertheless suggests that curbing nutrient input, though requiring considerable and growing effort in agricultural practices, will continue to pay off.
